# Intravenous ibuprofen versus diclofenac plus orphenadrine in orthognathic surgery: a prospective, randomized, double-blind, controlled clinical study

**DOI:** 10.1007/s00784-022-04381-5

**Published:** 2022-02-01

**Authors:** Josip Tomic, Jürgen Wallner, Irene Mischak, Gerald Sendlhofer, Wolfgang Zemann, Monika Schanbacher, Hamid Hassanzadeh, Andreas Sandner-Kiesling, Michael Payer, Tomislav A. Zrnc

**Affiliations:** 1 Department of Oral and Maxillofacial Surgery, University Hospital, Medical University of Graz, Auenbruggerplatz 5, 8036 Graz, , Styria Austria; 2 Department of Dental Medicine and Oral Health, University Hospital, Medical University of Graz, Auenbruggerplatz 5, 8036 Graz, , Styria Austria; 3 Executive Department for Quality and Risk ManagementDivision of Plastic, Aesthetic and Reconstructive SurgeryDepartment of Surgery, Research Unit for Safety in Health, Medical University of Graz, Graz, Austria; 4 Department of Orthopaedic Surgery, Johns Hopkins University, Baltimore, MD USA; 5 Department of Anaesthesiology and Intensive Care Medicine, Medical University of Graz, Graz, Styria Austria

**Keywords:** Analgesia, Numeric rating scale, Orthognathic surgery, Pain relief

## Abstract

**Objectives:**

The aim of this prospective, randomized, double-blind, controlled clinical study was to evaluate the analgesic effect of ibuprofen versus diclofenac plus orphenadrine on postoperative pain in orthognathic surgery.

**Material and methods:**

Patients who underwent orthognathic surgery were randomized into two groups to receive intravenously either 600 mg of ibuprofen (I-group) or 75 mg diclofenac plus 30 mg orphenadrine (D-group), both of which were given twice daily. Additionally, both groups were given metamizole 500 mg. Rescue pain medication consisted of acetaminophen 1000 mg and piritramide 7.5 mg as needed. To assess the pain intensity, the primary end point was the numeric rating scale (NRS) recorded over the course of the hospital stay three times daily for 3 days.

**Results:**

One hundred nine patients were enrolled (age range, 18 to 61 years) between May 2019 and November 2020. Forty-eight bilateral sagittal split osteotomies (BSSO) and 51 bimaxillary osteotomies (BIMAX) were performed. Surgical subgroup analysis found a significant higher mean NRS (2.73 vs.1.23) in the BIMAX D-group vs. I-group (*p* = 0.015) on the third postoperative day. Additionally, as the patient’s body mass index (BMI) increased, the mean NRS (*r* = 0.517, *p* = 0.001) also increased. No differences were found between age, gender, length of hospital stay, weight, operating times, number of patients with complete pain relief, acetaminophen or piritramide intake, and NRS values. No adverse events were observed.

**Conclusion:**

The results of this study demonstrate that ibuprofen administration and lower BMI were associated with less pain for patients who underwent bimaxillary osteotomy on the third postoperative day. Therefore, surgeons may prefer ibuprofen for more effective pain relief after orthognathic surgery.

**Clinical relevance:**

Ibuprofen differs from diclofenac plus orphenadrine in class and is a powerful analgetic after orthognathic surgery.

## Introduction

Orthognathic surgery is a standard procedure in maxillofacial surgery. The current concept of correcting facial asymmetry dates back to the 1950s, when Obwegeser began introducing the idea of splitting the mandible [1]. What became known as the “bilateral sagittal split osteotomy” (BSSO) is now the dominating technique to set back a protruding or set forward a retruding lower jaw. In addition, BSSO can be combined with the Le Fort I osteotomy to correct midfacial deformities [2, 3]. These cosmetic and corrective surgical procedures are associated with extensive soft tissue damage and swelling that may cause moderate-to-severe postoperative pain. [4]

Acute intense pain is not uncommon following orthognathic surgery, and untreated may lead to undue suffering among patients [5, 6, 7]. In addition, it is known that undermanaged pain results in delayed healing and recovery in the postoperative setting, which can furthermore interfere with patient’s daily activities [8, 9]. If pain control remains inadequate, a substantial proportion of patients continue to experience postsurgical pain and increased opioid consumption despite administration of multiple analgesics. Consequently, acute pain and its sequelae are the most common reason for hospitalization and emergency department visits following orthognathic surgery. [13]

Therefore, adequate pain management is vitally important to patient outcomes. Pain management is unique to each patient and dependent on multiple factors including the severity and temporality of the pain, as well as the patient’s physical function [8]. Current management of mild-to-moderate pain utilizes nonopioids treatments such as nonsteroidal anti-inflammatory drugs (NSAIDs) and acetaminophen. As with any medication, it is important to be aware of potential side effects, as for NSAIDS these include nausea, gastrointestinal bleeding, and cardiovascular events [8]. Similarly, acetaminophen is considered the safest analgetic agent but has a known hepatotoxic risk [8]. Metamizole is another potential nonopioid alternative, but its use is controversial and requires a warning about the risk of agranulocytosis. As pain severity increases, opioids become the drug of choice but should be taken for the shortest amount of time necessary. Common side effects of opioids include nausea, vomiting, constipation, and use disorder which are well documented in the medical literature. [10, 11, 12]

The US opioid crisis of recent years refocused clinicians’ interest in utilizing the combined use of NSAIDs along with several other nonopioid analgesic agents such as acetaminophen. As a consequence, opioid analgesics are recommended only if NSAIDs (e.g., diclofenac, ibuprofen) have proven ineffective [14]. Currently, clinical practice varies regarding the use of NSAIDs in combination with opioids for pain management in patients undergoing orthognathic surgery [15]. A recent randomized controlled study that used intravenous single-dose IV ibuprofen for preventive analgesia in comparison to saline showed both a reduction of pain scores and opioid consumption. [16] Various studies have highlighted the need for additional evidence related to outcomes for the optimal pain management following surgery [14, 17]. The present study is the first to compare ibuprofen vs. diclofenac plus orphenadrine in orthognathic surgery for the treatment of postoperative pain.

The aim of this study was to assess pain scores in individuals receiving either ibuprofen or diclofenac plus orphenadrine after orthognathic surgery.

## Material and methods

### Study design

A prospective, randomized, double-blind, controlled clinical trial was conducted with one group receiving intravenous ibuprofen 600 mg twice daily (I-group) or diclofenac 75 mg plus orphenadrine 30 mg twice daily (D-group). The study was conducted at the Division of Oral and Maxillofacial Surgery, at the Medical University of Graz, Austria.

### Population and sample

The sample was obtained from patients who underwent orthognathic surgery consisting of Le Fort I, bimaxillary or bilateral sagittal split osteotomies (BSSO), and segmental osteotomies. Additional surgical procedures were chin osteotomies. All patients stayed in the hospital for at least 3 days. Patients with surgically assisted rapid palatal expansion (SARPE) were discharged from the hospital after the latency period of 3 days, if they were able to activate the appliance (bone-borne or tooth-borne) to achieve adequate expansion (0.5 mm per day). Baseline measures, including personal data of all patients, demographic characteristics such as sex and age, and clinical data such as comorbidities, were collected. Data was obtained from both patients and their medical records. Eligible participants provided written informed consent and signed a patient release of information form to authorize publication of data.

### Inclusion and exclusion criteria

Inclusion to the study was limited to patients 18 years of age or older whom were undergoing orthognathic surgery. Indication for surgery was based on clinical findings and cephalometric analysis after consultation of a maxillofacial surgeon and an orthodontist. Exclusion criteria were as follows: any additional simultaneous operative nose or eye lid procedures, use of antidepressant agents, chronic pain syndrome, baseline pain at admission (from any disease but temporomandibular joint disease), cognitive impairment, syndromes, allergy, and intolerance or contraindication to the investigational medications. Additionally, pregnant or breast feeding patients were excluded from the study.

### Surgical procedures

All surgical procedures took place in the hospital and were performed by a single surgical team. The same surgical protocol was followed. All patients received general anesthesia.

Administration of analgesic medications during surgery were performed by an anesthesiologist and consisted of either intravenous ibuprofen 600 mg or diclofenac 75 mg plus orphenadrine according per the study protocol. Additionally, metamizole 1 g and piritramide 3.75 mg was given intravenously as needed.

Before incision, local anesthesia was administered (articaine 4% with epinephrine 1:100.000, Ultracaine; Sanofi-Aventis, Bridgewater, NJ, USA). Mepivacaine HCl 4% (Scandicaine; AstraZeneca, Wedel, Germany) was administered in case of articaine or epinephrine contraindications. One dose of systemic glucocorticoids (intravenous prednisolone 1 mg per kg per day) was administered during the operation.

BSSO was performed through a buccal osteotomy of the mandibular body. Medial ramus osteotomy started at the ridge and terminated just posterior to the lingual into the retrolingual fossa. Then the sagittal osteotomy was made through the buccal cortex of the mandibular ramus and body. Lingual and buccal cortical plates were separated. A splint was used, and the planned occlusion was secured with holding wires. Three screws were used for the rigid fixation through a transbuccal approach on each side.

For two jaw surgery, Le Fort I osteotomies were undertaken through a horizontal osteotomy line 5 mm above the root apices. The osteotomy was made from the pyriform rim to the posterior maxilla. After downfracture, the maxilla was passively repositioned using an interim splint and holding wires. Internal fixation was then applied with four plates, and the interim splint was removed. Then BSSO was performed as described above. Elastics were routinely applied to all patients for 2 weeks.

### Pain control after surgery

Study participants received either ibuprofen 600 mg or diclofenac 75 mg with orphenadrine 30 mg intravenously twice per day for 3 days to match the dosing regimens of both arms. The maximum daily adult dose of ibuprofen (Ibuprofen B. Braun 600 mg) is 1200 mg and of diclofenac 150 mg (Neodolpasse® Fresenius Kabi Austria GmbH) for intravenous application respecting the instructions given by the manufacturers and the anesthesiologist at the Medical University of Graz. From the fourth postoperative day until discharge, oral analgesics (either ibuprofen 600 mg or diclofenac 50 mg twice per day respectively) were administered without change to treatment arm.. For better pain relief, all patients received metamizole 500 mg three times per day. Rescue medication consisted of acetaminophen 1000 mg up to three times a day. If patients had not experienced sufficient pain relief, piritramide 7.5 mg was administered as needed up to four times a day. Investigational medication was not blinded. Participants in both groups were instructed to use local ice application. Moreover, a liquid diet was introduced after surgery.

### Simple randomization and blinding procedures

For bias reduction, the treatment allocation was performed double-blinded and randomized controlled. Simple (unrestricted) randomization was performed intraoperatively in consecutive alternation by one consistent operating team for all orthognathic surgery cases.

Patients who met all inclusion criteria were randomly assigned to a randomization identification code: I- or L-group. Randomization was performed preoperatively in the OR by the single orthognathic operating team. As per the protocol, each operative case alternated pain management; thus, the first case was treated with ibuprofen, the next diclofenac plus orphenadrine, and then the following with ibuprofen. Additionally, investigators (*n* = 3), participants (109), and outcome assessors (*n* = 2) were blinded to the group allocation. Randomization and blinding were not disclosed to the participants, outcome assessors, or investigators until the end of the trial. There was an independent doctor supervising the pain medication.

### Study variables

The primary outcomes assessed in the present study were the postoperative patient numeric rating scale (NRS) pain scores. Postoperative pain assessments started on the day of surgery and continued every 6 to 8 h, three times a day over the course of the hospital stay. The first reported NRS scores were recorded 4 h after surgery on postoperative day (POD) 0. NRS scores for POD 1–3 are reported, considering three scores were measured throughout each postoperative day, and only 1 score is reported per POD; thus, the scores were averaged across each POD. Additionally, a 24 h NRS ≤ 1 is defined as a period without pain or close to no pain for 24 h.

Prespecified secondary outcomes included the total opioid and acetaminophen use. In addition, the incidence of major adverse events from pain medication (myocardial infarction, stroke, and cardiovascular mortality), major postoperative complications (relapse, severe bleeding, bad split), BMI, body weight (kilograms), demographic data (sex, age), and average length of hospital stay (days) were recorded.

### Statistical analysis

#### Sample calculation

An independent statistician conducted the data analysis. It was estimated that a sample of 35 patients in each group would provide the trial with 80% power to detect a difference in mean NRS (the difference between a group L mean, µ_1_, of 15.8 and a group C mean, µ_2_, of 19.2) assuming that the common standard deviation is 5.0 using a two sample *t* test with a 5% two-sided significance level. [18]

### Analysis of outcome measures

A general linear model (GLM) with repeated measurements was used to assess the mean NRS across the study period on POD-0, POD-1, POD-2, and POD-3. A Mann–Whitney *U* test was used to analyze the 24 h NRS ≤ 1.

Pearson coefficients (r) were computed to test correlations and associations between the study variables (acetaminophen, piritramide intake, age, length of stay calculated in days from admission to discharge, body weight, BMI, and duration of surgical procedure in both groups). The correlation coefficient according to Pearson takes values between −1 and 1.

The *t* test was used to determine whether there was a significant association between gender and mean NRS values among the D-group and I-group. Subgroup analysis included 4 subgroups with consideration to the two most commonly performed surgical procedure: BIMAX and BSSO.

Significant differences were accepted if *P* ≤ 0.05. Statistical analyses were performed using SPSS version 25.0 (IBM SPSS Statistics for Windows, Armonk, NY: IBM).

## Results

**Figure 1:**
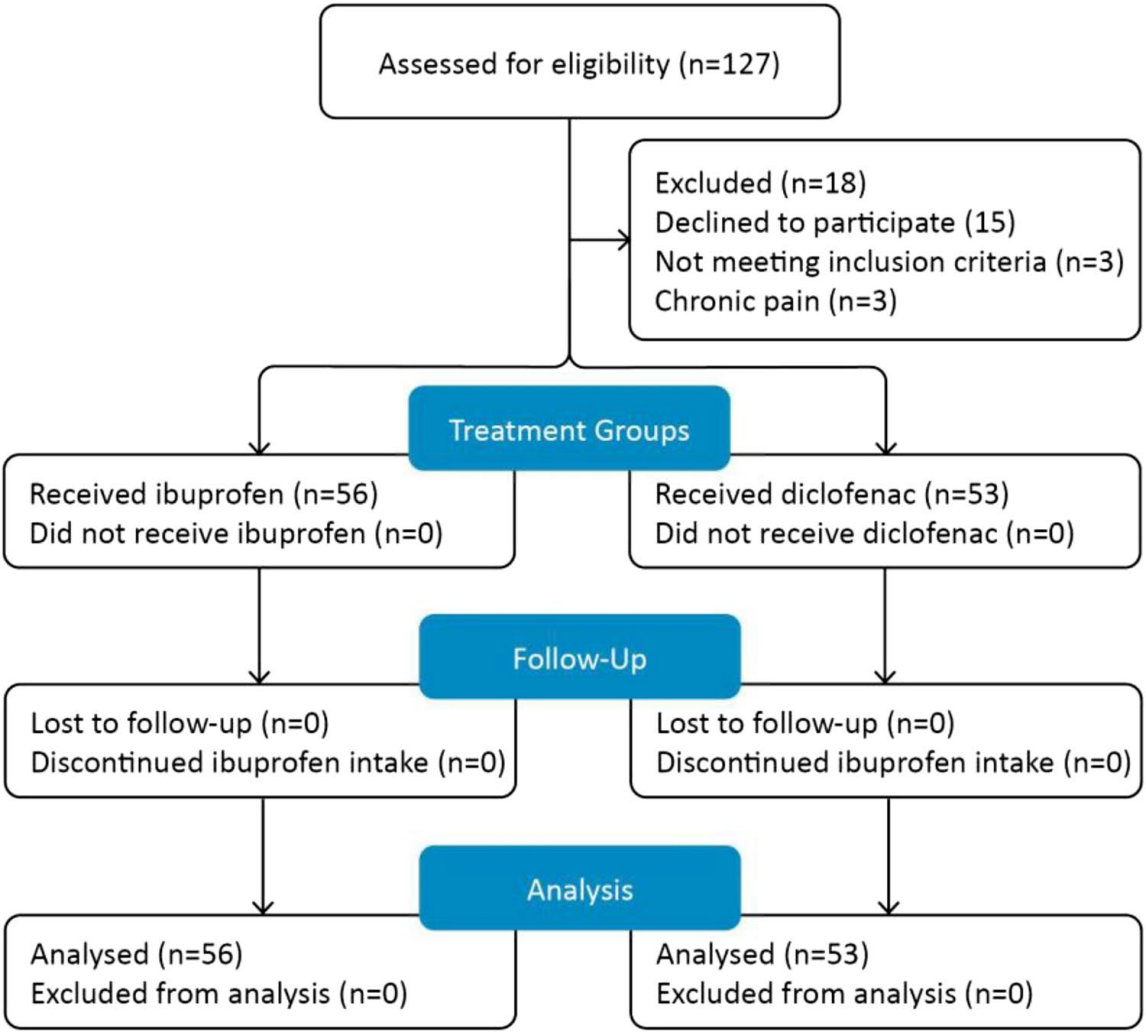
Enrollment of the study population

Fifty-six patients were recruited to I-group (ibuprofen) and 53 to D-group (diclofenac). No one was excluded from this study (Fig. 1). The two groups (ibuprofen vs. diclofenac plus orphenadrine) were similar with respect to their demographic characteristics and pre-existing comorbidities. Out of 109 participants, 65 were female (59.63%) and 44 were male (40.36%) with a mean age of 28.0 and 28.4 years, respectively (*p* = 0.759). There were no significant differences (*p* = 0.987) with respect to comorbidity status across cohorts. The most common comorbidities were smoking (10.09%, *n* = 11), followed by anemia (1.83%, *n* = 2), diabetes mellitus (1.83%, *n* = 2), hypertension (1.83%, *n* = 2), thrombocytosis (1.83%, *n* = 2), and bronchial asthma (1.83%, *n* = 2).

### Primary outcome measures

**Figure 2:**
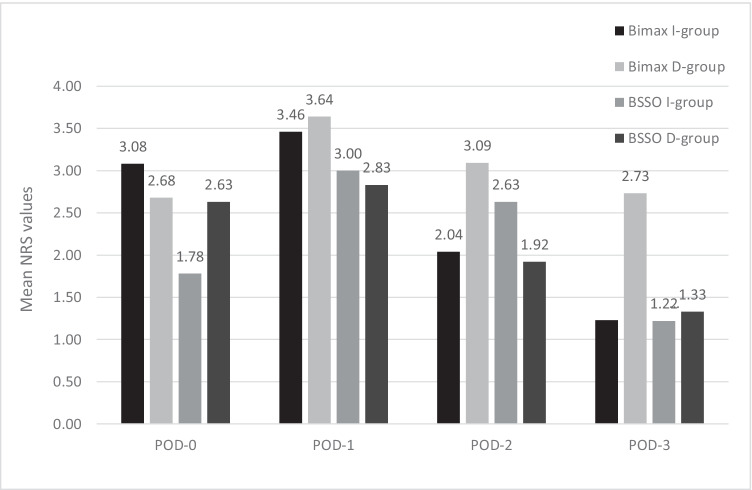
Comparison of pain scores between the ibuprofen and diclofenac groups. BIMAX, bimaxillary; BSSO, bilateral sagittal split osteotomies; D-group, diclofenac group; h, hours; I-group, ibuprofen group; NRS, numeric rating scale; POD, postoperative day. The asterisk indicates *p* < 0.05 (seen only in the Bimax I-group at POD-3).

The GLM repeated measurement analysis showed a significant difference in NRS pain scores over 3 consecutive days for both groups (*p* < 0.001), though the difference between the groups was not significant (*p* = 0.352). Figure 2 shows the change of the NRS values over the study period. The mean NRS pain score peaked on day 1 and began to showed a decline by day 2.

The mean NRS on the day of surgery was 2.58 (95 % confidence interval [CI], 1.94 to 3.23) for patients who received diclofenac plus orphenadrine vs. 2.39 (95% CI, 1.84 to 2.94) for patients who received ibuprofen (*p* = 0.649). Similarly, the mean NRS on the first postoperative day was 3.06 (95% CI, 2.44 to 3.67) for D-group vs. 3.16 (95% CI, 2.52–3.80) for I-group (*p* = 0.814). The mean NRS on the second postoperative day was 2.45 (95% CI, 1.81–3.09) for the D-group vs. 2.36 (95% CI, 1.78-2.94) for I-group (*p* = 0.825). The mean NRS on the third postoperative day was 1.89 (95% CI, 1.25–2.52) for D-group vs. 1.23 (95% CI, 0.74–1.73) for I-group (*p* = 0.104). The orthognathic surgical procedures performed are listed in Table 1.

**Table 1:** Distribution of the performed orthognathic surgical procedures

	**Total**		**Diclofenac group**		**Ibuprofen group**		**Significance**
	***n***	**%**	***n***	**%**	***n***	**%**	
**BIMAX**	48	44.0	22	41.5	26	46.4	NS
**BSSO**	51	46.8	24	45.3	27	48.2	NS
**Le Fort I**	4	3.7	4	7.5			NS
**SARPE**	5	4.6	2	3.8	3	5.4	NS
**Segmental**	1	0.9	1	1.9			NS

*BIMAX*, bimaxillary osteotomy; *BSSO*, bilateral sagittal split osteotomy; *NS*, no significance (*p* > 0.05); *SARPE*, surgically assisted rapid palatal expansion; segmental, segmental osteotomy

### Subgroup analysis

Analysis of four surgical subgroups (BIMAX I-group with 26 patients vs. BIMAX D-group with 22 patients and BSSO I-group with 27 patients vs. BSSO D-group with 24 patients) revealed no differences with the use of an independent sample *t* test, for mean NRS on POD-0, POD-1, and POD-2, respectively.

In contrast, the mean NRS POD-3 was significantly decreased in the BIMAX I-group with a mean of 1.23 compared to the BIMAX D-group 2.73 (*p* = 0.015) between the BIMAX D-group Additional subgroup analysis is provided in Figure 2.

Using a Mann–Whitney *U* test, the 24 h NRS scores of ≤ 1 showed no significance (*p* = 0.549) between groups. These pain-free or close-to-pain-free periods occurred in 12.50% (7 of 56 patients) in the I-group 16.98% (9 of 53 patients) and in the D-group.

### Secondary outcome measures

Secondary outcome variables (age, gender, length of stay, weight, and length of operating times) were not associated with higher NRS values at the study time points (*p* > 0.05). The average length of stay (LOS) for patients who received diclofenac plus orphenadrine was 6.21 days ± 1.12 (range, 4 to 9 days) and for patients who received ibuprofen 6.16 days ± 0.87 (range, 5 to 8 days) (*p* = 0.807).

The mean operating times varied among procedures . Bimaxillary procedures had the longest operative time lasting on average 123.50 min. Segmental osteotomy was the shortest, lasting only 18 min. BSSO on average took 68.44 min. Finally, the Le Fort I osteotomies averaged 90.40 min.

Higher BMI was associated with higher NRS on the second and third postoperative day for the D-group (*p* = 0.015 and *p* = 0.001, respectively) and for the I-group (*p* = 0.038 and *p* = 0.005, respectively).

Total breakthrough pain acetaminophen use was increased in the I-group (*n* = 46 D-group, *n* = 78 for the I-group) (*p* = 0.006). Piritramide intake was similar across cohorts, (*n* = 41 for the D-group and *n* = 31 for the I-group) (*p* = 0.779). The frequency of chin osteotomies (14.67%, *n* = 16) did not differ (*p* = 0.654) between groups.

No major postoperative complication or adverse events due to pain medication were observed.

## Discussion

The present study chose to assess the combination of diclofenac and orphenadrine together as a postoperative analgesic due to its ready-to-use infusion, current lack of alternatives to ibuprofen, and previously proven efficacy when used in combination [19]. This randomized controlled clinical study found the analgesic effect of ibuprofen superior to diclofenac and orphenadrine following bimaxillary osteotomy as evidenced by the higher mean NRS pain scores in the D-group on postoperative day 3. Additionally, it was noted that as a patient’s body mass index (BMI) increased, the postoperative mean NRS also increased.

Orthognathic surgery addresses both esthetic and functional problems by mobilizing the mandible and/ or the maxilla, which unfortunately can lead to substantially increased morbidity due to postoperative pain [16, 4, 8]. Modern pain management strategies build on opioids, steroids, nonpharmacologic methods, and nonsteroidal anti-inflammatory drugs (NSAIDs) [16]. Following orthognathic surgery, NSAIDs are the most commonly prescribed medication for postoperative pain medications [17]; however, the lack of evidence regarding pain management in oral and maxillofacial surgery results in wide variations in analgesic practice. [18, 20, 20]

Of importance, a consensus has emerged in cardiovascular pharmacotherapy regarding diclofenac, noting it has the potential for higher rates of cardiovascular side effects. [24] The European Medical Agency has released two Pharmacovigilance Risk Assessment Committee (PRAC) recommendations on diclofenac (2013) and ibuprofen (2016) to increase awareness of potential cardio- or cerebrovascular side effects while still confirming that both drugs are valuable analgesics in the appropriate patients [21, 22]. Following these PRAC recommendations, patients with pre-existing cardiovascular risk factors should receive naproxen as an alternative pain management strategy, although it may induce similar complications. [21, 22] Alternatively, both cardiologists and pharmacologists have advocated for acetaminophen or metamizole use for mild-to-moderate pain, with additional escalation to opioids if patients do not achieve satisfying pain relief [23]. Interestingly, to date, no studies have reported the supplemental use of metamizole in orthognathic surgery. This could in part be due to the drug’s safety profile. While metamizole is an effective analgesic and is broadly used in many European countries, it possesses a varying risk of fatal agranulocytosis and hepatotoxicity [24]. Thus, there is still considerable controversy surrounding its use. In the present study, the authors found no adverse events from metamizole. [18, 20–22, 23]

A review and position paper by the working group for Cardiovascular Pharmacotherapy of the European Society of Cardiology, the number needed to harm for diclofenac, because of a serious cardiovascular events, was 25. [25] For example, a low NNH indicates that one is more likely to encounter the harm than the benefit, as a serious adverse event is encountered after treating just 25 patients. Initially, it was expected that patient who received diclofenac plus orphenadrine would have a higher incidence for serious medication-related adverse events. However, no serious adverse events from pain medication occurred in the present study population (*n* = 109). Our assumption was that this patients are of relatively young age and therefore are less prone to cardiovascular problems. Definitely, the question of whether certain NSAIDs have advantages over others has not been settled yet.

The results of the present study showed a significant pain reduction in the bimaxillary subgroup treated with ibuprofen, in contrast to no differences for the BSSO subgroup treated with ibuprofen. This could be explained by the more invasive nature of the bimaxillary procedure and the longer operating times. In addition, complication rates were reported to be higher among patients undergoing bimaxillary surgery in the literature. [26] In both groups, the NRS scores peaked on day 1 and decreased on day 2.

The authors did not encounter any major complications in the present study. Common minor surgical complications included swelling and impaired mouth opening. The potential association of postoperative swelling and pain remains unclear. Several NSAIDs used for the treatment of acute postoperative pain in maxillofacial surgery have demonstrated ability to reduce edema, as well as anti-inflammatory properties. However, literature suggests that there is no association between the degree of facial edema and pain severity, although it is difficult to quantify swelling [27, 28]. The current proposed methods for measuring the degree of soft tissue swelling include CT and stereophotogrammetry. Though CT was reliable, it exposed patients to unnecessary radiation [29]. Interestingly, three-dimensional stereophotogrammetry showed patients with a higher BMI had the greatest amount of swelling. [30]

Secondary outcomes did not generally favor ibuprofen over diclofenac plus orphenadrine; however, an association of high BMI and high NRS scores was found in both groups. In addition, the results of this study are in agreement with previous research which has found no significant between groups with respect to age, gender, weight, and duration of operation [31, 32]. Interestingly, the present study showed an association between higher BMI and pain intensity across all patients.

Moreover, the present study investigated the patient-centered use of opioids, since NSAIDs are known to have an opioid sparing effect [33]. The opioid consumption was not significantly different between the groups. Although patients who receive opioids carry the risk of overdose and use disorder, opioids are often necessary for adequate analgesia [34]. The potential importance of opioids is supported by previous observations, with the combination of opioids and NSAIDs being the best treatment for acute postoperative pain in maxillofacial surgery. Therefore, brief opioid courses rather than NSAIDs alone are the preferred therapy for severe pain. [35]

The patient discharge decisions may be complex and complications are not predictable. Improving quality of care and simultaneously lowering costs are challenges for the healthcare system in addition to preventing unnecessary readmissions. At the investigators’ institution, patients were kept for at least 3 days in the hospital. The mean LOS for all procedures was 6.18 days (range 4 to 9 days), which falls within the range of 1.7 to 20.0 days reported in the literature [36]. Different global discharge patterns were attributed to different management practices, insurance coverage, infrastructure (large-bed vs. small-bed hospitals, outpatient nursing facilities), quality of care, and hospital characteristics (teaching vs. nonteaching hospitals).^37^Several limitations must be acknowledged, including the fact that study group assignment did not undergo block or stratified randomization; thus, selection bias cannot be ruled out. In addition, a placebo group was not included to compare the study regimen due to ethical concerns about administering placebo in patients with acute severe pain. The postoperative swelling was not quantified, likely to be greater in patients with bimaxillary osteotomies. Additionally, the study did not provide information on the anti-edema activity of steroids on postoperative pain. Given the young study population and lack of comorbidities, the study results may not be generalizable to patients with coexisting conditions (e.g., cardiovascular disease) and older patients (> 60 years of age).

The present study demonstrated that ibuprofen is more effective than diclofenac plus orphenadrine in bimaxillary cases providing greater postoperative pain relief. Additionally, the study found increased BMI is associated with higher pain scores following bimaxillary surgery, yet, age, gender, and operating time did not significantly impact postoperative pain.
